# Effects of Modified Handling on the Physiological Stress of Trawled-and-Discarded Yellowfin Bream (*Acanthopagrus australis*)

**DOI:** 10.1371/journal.pone.0131109

**Published:** 2015-06-22

**Authors:** Sven Sebastian Uhlmann, Matt Kenyon Broadhurst, Russell Brian Millar

**Affiliations:** 1 School of Environmental Sciences and Natural Resources Management, National Marine Science Centre, University of New England, Coffs Harbour, New South Wales, Australia; 2 Department of Primary Industries, Fisheries Conservation Technology Unit, Coffs Harbour, New South Wales, Australia; 3 Marine and Estuarine Ecology Unit, School of Biological Sciences, University of Queensland, Brisbane, Queensland, Australia; 4 Department of Statistics, The University of Auckland, Auckland, New Zealand; James Cook University, AUSTRALIA

## Abstract

Modified handling is often claimed to reduce (sub-)lethal impacts among organisms caught-and-released in fisheries. Improving welfare of discarded fish warrants investigation, when their survival is of both economic and ecological importance. In this study, juvenile yellowfin bream (*Acanthopagrus australis*) were trawled in an Australian penaeid fishery and then discarded after on-board sorting in either dry or water-filled (modified) trays and with delays in starting sorting of either 2 or 15 mins. Blood plasma cortisol, glucose and potassium were sampled immediately from some yellowfin bream, while others were placed into cages (with controls) and sampled after five days. Irrespective of their on-board handling, all trawled fish incurred a relatively high acute stress response (i.e. an increase in Mean ± SE cortisol from a baseline of <4 to 122.0 ± 14.9 ng/mL) that was mostly attributed to the trawling process, and exacerbated by variation in key parameters (low salinity, changes in water temperature and the presence of jellyfish *Catostylus mosaicus* in catches). When *C*. *mosaicus* was present, the potassium concentrations of fish sampled immediately after sorting were significantly elevated, possibly due to nematocyst contact and subsequent inhibition of ion pumps or cytolysis. Stress also increased during handling in response to warmer air temperatures and longer exposure. While most fish had substantially recovered by 120 hours after discarding, deploying selective trawls (to reduce jellyfish) for short periods and then quickly sorting catches in water would benefit discard welfare.

## Introduction

The discarding of unwanted organisms during fishing is perceived as ecologically and economically wasteful; particularly if most discards die [1]. Beyond immediate mortalities are potentially deleterious physiological effects that may impair the fitness of surviving individuals by compromising their health, growth and reproductive output [1–6]. Some of these impacts may be reduced via more benign on-board handling practices, such as minimising air exposure among teleosts [7, 8]; the benefits of which have been demonstrated for several species after capture by passive gears such as hook-and-line [9]. It is less clear whether such practices have utility in alleviating stress among fish caught by mobile gears—although benefits have been realised for crustaceans [10–13].

During trawling, teleosts often are severely and acutely stressed through exercise and interactions with netting panels and each other [14]. Cumulative stress is then evoked as catches are dropped on deck and exposed to air, sunlight and temperature differentials while being handled [15–17]. Teleosts respond to such stressors through various biochemical and physiological processes that manifest as measurable changes to their blood chemistry [18]. Specifically, rapid increases in plasma catecholamines by chromaffin cells result in elevated plasma cortisol as a primary response, with secondary effects including changes in glucose [19, 20] and hydrominerals such as potassium due to electrolytic disturbances [18, 21]. All of these responses may be compounded by prolonged exposure to air, thermal or haline shock [16, 20]. As one example, among some pink snapper (*Pagrus auratus*), acute capture and handling stress disrupted endocrine reproductive processes, including decreased gonadal steroid concentrations or gamete quality [22].

While varying physiological responses to trawling-related stressors are inevitable, limiting or resolving the build-up of primary indicators and secondary metabolites in the blood and restoring energy deposits in the muscles after exhaustion may reduce chronic symptoms [2]. In particular, such outcomes can be achieved in some species by minimizing hypoxia and severe temperature changes [16, 17, 23, 24]. For example, rapidly sorting catches in water or allowing for their recovery prior to their release and limiting air exposure, may represent promising approaches [7, 8].

Nevertheless, sensitivity towards capture-related stress is species-specific and depends on the severity and duration of stressors, anatomical and physiological characteristics and ecological factors [5, 9]. Some species may survive at a high rate after discarding, but could nevertheless incur physiological disturbances with unknown protracted consequences. Yellowfin bream (*Acanthopagrus australis*) is one species for which there are such concerns. It is highly valued by both commercial and recreational fishers in south-eastern Australia, and apparently well-adapted to tolerating various anthropogenic impacts, including discarding [7, 23, 25–30]. For example, in a recent study quantifying the discard mortality and key contributing factors from Australian estuarine penaeid trawls, fatalities among yellowfin bream discarded into floating cages remained below 50% within 120 hours, although there were slight ongoing cumulative deaths depending on initial treatments [7]. Key factors affecting mortality included the total length (TL) of fish, sorting under dry conditions, a protracted delay in starting sorting within conventional periods, the weight of jellyfish (*Catostylus mosaicus*) in the catch and salinity [7]. Simply sorting yellowfin bream in a water-filled container reduced mortalities by up to a quarter, indicating the utility of reducing air exposure during on-board handling [7].

While these results were positive from a conservation perspective, attempting to map the physiological responses (and any explanatory factors) of yellowfin bream to modified on-board handling procedures may not only provide more insight into the utility of alternative catch-processing techniques, but also the consequences for surviving individuals. Considering the above, our main aim here was to quantify the stress response and key contributing factors among trawled yellowfin bream (i) immediately after on-board handling across a range of conceivable handling conditions including dry and modified, wet sorting and then (ii) five days after discarding. We hypothesised that modified handling with minimal air exposure would alleviate physiological stress responses to hypoxia, but that the presence of jellyfish would have deleterious consequences. Our physiological analyses were restricted to cortisol and glucose considering their proven utility in other stress studies [2, 18], and potassium due to its sensitivity to hypoxia [16] and marine biotoxins [31]. A secondary aim of the study was to use the results to suggest simple applied strategies to minimise negative physiological responses, and eventually maximise yellowfin bream welfare.

## Materials and Methods

### Ethics statement

This research was done in the Clarence River (29°27’S, 153°09’E) New South Wales (NSW) Australia in accordance with the Department of Primary Industries scientific collection permit (No. P01/0059A-2.0). No specific permissions were required to access the Clarence River and charter a commercial vessel—other than internal contractual agreements. This study did not involve endangered or protected species. Animal ethics approval was granted for this research by the NSW DPI Animal Care and Ethics Committee (ACEC REF 05/10).

### Equipment and treatments

The study was done during the austral summer 2007 in the Clarence River (29°27’S, 153°09’E) using a commercial penaeid trawler (10 m in length) towing double-rigged trawls (7.4-m headlines and 40 mm stretched mesh opening—SMO) attached to square-mesh codends (27 mm SMO on the bar; see [7] for a gear description and Table 1 for the experimental design). A Greenspan EC350 data logger was attached to one of the trawls (to record water temperature and salinity—see below) and both were deployed for 30 min, after which the codends were emptied into an 810-L sorting tray that was alternatively (i) left empty (conventional handling), or (ii) filled with river water (modified handling)—via a flow-through system and monitored for dissolved oxygen (DO), temperature and salinity using a hand-held Horiba. Within each sorting-tray configuration, the catches were left for either (i) 2 or (ii) 15 min before starting sorting (i.e. the range of conventional sorting delays). During sorting, live yellowfin bream were removed after treatment and some sampled immediately for blood by a researcher (T_0_; see below), while another researcher placed other fish in groups of five into water-filled, aerated 100-L cages that were transported on board a dory to a nearby monitoring site and submerged (following [7]).

**Table 1:** Number of yellowfin bream per deployment from which blood plasma samples were obtained at T_0_ and T_120_ in each treatment group (2- or 15-min delay in starting either conventional or modified sorting).

Date	Sorting delay	Sorting method	Deployment	T_0_	T_120_
03/15/07	15 min				
		Control	-	n/a	3
		Conventional	1	3	3
		Conventional	3	2	n/a
		Modified	2	2	1
		Modified	4	n/a	2
03/16/07	2 min				
		Control	-	n/a	n/a
		Conventional	6	2	n/a
		Conventional	8	3	n/a
		Modified	5	2	n/a
		Modified	7	n/a	n/a
03/20/07	2 min				
		Control	-	n/a	4
		Conventional	10	2	1
		Conventional	12	1	5
		Modified	9	n/a	2
		Modified	11	n/a	2
03/21/07	15 min				
		Control	-	n/a	2
		Conventional	2	1	1
		Conventional	4	4	7
		Modified	1	1	n/a
		Modified	3	1	n/a
03/22/07	2 min				
		Control	-	n/a	6
		Conventional	5	n/a	6
		Conventional	7	2	1
		Modified	6	n/a	1
		Modified	8	2	n/a
04/18/07	2 min				
		Control	-	n/a	8
		Conventional	2	3	3
		Conventional	4	3	3
		Modified	1	3	4
		Modified	3	n/a	n/a
04/19/07	15 min				
		Control	-	n/a	8
		Conventional	5	3	4
		Conventional	7	3	4
		Modified	6	3	1
		Modified	8	3	2
04/26/07	2 min				
		Control	-	n/a	8
		Conventional	2	2	1
		Conventional	4	3	3
		Modified	1	3	6
		Modified	3	n/a	2
04/27/07	15 min				
		Control	-	n/a	7
		Conventional	6	2	4
		Conventional	8	1	6
		Modified	5	3	5
		Modified	7	1	2

Control fish were sampled at T_120_.

n/a, insufficient fish for sampling; -, not applicable.

Appropriate numbers of control yellowfin bream were also sampled during the experiment. These fish were originally caught using non-invasive methods [30], and acclimated for at least one week in six 3000-L aerated holding tanks (with flow-through seawater at 63 L/min and <5 kg/m^3^ stocking density) located on the banks of the Clarence River and tributaries, and adjacent to the trawled area to ensure identical water quality To minimise stress and damage to the control yellowfin bream during transfer (and following animal ethics requirements), they were anaesthetised using between 25 and 35 mg/L of benzocaine (ethyl-p-amino benzoate). Randomly selected, sedated control fish were scooped from the holding tanks with knotless fine-meshed nets into 5-L PVC buckets and placed into the same water-filled and aerated cages used for the trawled-and-discarded fish on-board the transporting dory, before being transferred the same distance to the monitoring site and secured there [7].

All separately caged control and treatment fish and their immediate environment (DO, temperature and salinity) were monitored every 24 hours up until 120 hours, after which live individuals were randomly selected, removed and sampled for blood (T_120_; as below). Fish were not fed during their monitoring.

### Blood samples

At both T_0_ and T_120_, relevant yellowfin bream were dorsally secured inside a foam block and sampled with heparinised syringes (22-gauge needles) extracting blood from a caudal puncture within 1 min of handling (Table 1). Approximately 5 mL of blood was drawn into an Eppendorf vial, placed on ice and, within 30 min, centrifuged for 3 min at 5000 rotations per minute. All valid plasma samples were returned to the laboratory on the same day and frozen at -18°C. Within 60 days, the samples were packed in dry ice and sent to the Symbion Vetnostics laboratory in Traralgon, Victoria, and analysed for concentrations of: (i) cortisol (ng/mL) using a Siemens healthcare diagnostics kit and a Centaur analyser with chemi-luminescence; (ii) glucose (mmol/L) by colorimetric clinical kits (Roche Diagnostics, USA) and spectrophotometric assays; and (iii) potassium (mmol/L) via a Hitachi analyser using indirect ion-specific electrode measurements. Cortisol at concentrations <3.6 ng/mL were censored (at 3.6 ng/mL) in the pathology lab reports by Gippsland Vetnostics (*n* = 1 sample from T_0_, and 7 treatments and 18 controls from T_120_).

After bleeding, fish were measured to the nearest 0.5 cm TL before being released. Baseline estimates of cortisol and glucose from local wild populations of yellowfin bream were derived from previous studies at Means (± SE) of 3.6 ± 2.0 ng/mL and between 1.3 ± 0.4 and 2.5 ± 0.2 ng/mL, respectively [31, 32].

### Data and analyses

The following environmental and biological data were recorded for use as explanatory variables in the analyses: sampling time (T_0_ or T_120_); the sorting method (conventional or modified); delay in the start of sorting (2 or 15 min); temperature and salinity of the river at the bottom; percentage cloud cover; air temperature; the weight of jellyfish; air exposure time (min) and TL (cm). Additional measurements included technical deployment-specific variables (speed, depth and duration), and total catch, temperature and salinity at the river surface, and the concentration of DO in the water-filled sorting tray during modified sorting. These additional variables were not used in the analysis because they were either not of *apriori* interest, or were collinear with other variables, and in the case of DO because this measurement was only relevant for the modified sorting treatments.

The explanatory values were used in linear mixed models (LMMs) fitted to the analyte measurement on each fish. To avoid pseudo-replication [32] arising from lack of independence within individual deployments, the LMMs included haul (nested within day) as a random effect. The fits were obtained using the lmer function in the lme4 package of the R language (freely available from http://cran.stat.auckland.ac.nz/).

Three LMMs were applied to the trawled-and-discarded yellowfin bream; the first to all fish with sampling time (T_0_ vs T_120_) included as a factor; and the second and third LMMs to just those fish at either T_0_ or T_120_. A fourth LMM was also restricted to T_120_, but in addition to trawled fish it included the controls. For each analysis a backward variable elimination procedure was used, starting from an initial fit that included all potential terms of interest. For the first LMM, the initial fit included the three-way interaction of sorting method, delay and sample time, and two-way interactions of method and air exposure time, and method and air temperature, and the main effects of the other explanatory terms noted above. The second and third LMMs did not contain sample time terms. The fourth LMM included control fish and hence did not utilize any haul-specific variables, leaving only sampling method (conventional, modified, or control) and TL as the explanatory variables, and day as the sole random effect. At each step of the backward elimination, the least significant term was removed, continuing until only terms that were significant at the 0.05 level remained.

## Results

One-hundred-and-ninety-two yellowfin bream were sampled for blood (including 64 and 82 discarded individuals sampled at T_0_ and T_120_, respectively during nine days of trawling with four sampled hauls per day, and 46 controls at T_120_; Tables 1 and 2, and S1 Dataset). The former individuals were distributed among the four treatments of primary interest, including dry sorting after 2 (21 and 23 fish at T_0_ and T_120_, respectively) and 15 min (19 and 29), and wet sorting after 2 (10 and 17) and 15 min (14 and 13) (Table 1, S1 Dataset). During some trawls insufficient numbers were caught for blood sampling (Table 1).

**Table 2:** Summary of Mean (± SD) key environmental, technical and biological variables collected during the deployments.

Variable	Mean ± SD	*n*
**Technical variables**		
Deployment		
Speed (m/s)	2.7 ± 0.5	34
Depth (m)	4.3 ± 3.1	34
Duration (min)	30.7 ± 2.8	34
**Environmental variables**		
River temperature (o C)		
Surface	25.3 ± 1.9	34
Bottom	25.2 ± 1.9	34
River salinity		
Surface	9.9 ± 6.4	34
Bottom	11.3 ± 7.8	34
Cloud cover (%)	52.2 ± 35.4	34
Air temperature (o C)	23.0 ± 3.4	34
Water tray dissolved oxygen (mg/L)	4.8 ± 1.1	14
Water tray temperature (o C)	24.9 ± 1.9	16
Catch air exposure (min)		
2-min dry tray	3.7 ± 0.8	10
15-min dry tray	18.1 ± 1.6	8
2-min wet tray	1.9 ± 0.6	8
15-min wet tray	2.5 ± 0.8	8
**Biological variables**		
Total catch (kg)	19.4 ± 11.6	34
Jellyfish (kg)	1.1 ± 4.4	34
Trawled yellowfin bream total length (cm)	19.0 ± 3.5	146
Control yellowfin bream total length (cm)	19.7 ± 4.9	46

*n*, number or replicates.

The various environmental and technical parameters remained fairly consistent among replicate deployments, except for total catch and jellyfish weights and % cloud cover (Table 2). During the first two days of fishing, jellyfish were caught in three deployments (totals of 1.6, 12.7, 22.5kg); all of which were conventionally sorted (two after a 2-min delay and the other after 15 min).

Environmental parameters at the monitoring site were well within the preferred tolerance ranges of yellowfin bream, with the Mean ± SD temperature (24.9 ± 2.1°C) and DO (5.9 ± 0.9 mg/L) comparable to those during trawling. However, on average, the downstream monitoring site was more saline (36.6 ± 12.6) than the river (Table 2). Besides the effects of changing temperatures and salinities, none of the physiological correlates were affected by the delay in starting sorting or the modified handling in a water-filled tray.

### Cortisol

For the LMM applied to all trawled-and-discarded yellowfin bream across both sample times, significant effects were limited to fish at T_0_ having more than double the Mean ± SE plasma cortisol (122.0 ± 14.9 ng/mL) than those at T_120_ (55.3 ± 8.1 ng/mL), while irrespective of all other variables there was a negative association between cortisol and bottom salinity (*p* < 0.05; Table 3). No variables were significant for the LMM applied to T_0_ fish, but like the first model, salinity manifested as a significant predictor of variability in cortisol among T_120_ fish (*p* < 0.05; Table 3). The LMM assessing all fish at T_120_ detected a significant main effect of the treatment of fish, caused by controls having mean plasma cortisol concentrations (23.3 ± 5.7 ng/mL) that were lower than either dry- (51.6 ± 10.1 ng/mL) or wet-sorted (61.0 ± 13.1 ng/mL) yellowfin bream (*p* < 0.05; Table 3). The latter two groups were not significantly different (LMM, *p* > 0.05).

**Table 3:** Summary of variables (fixed effects) tested in linear mixed models for their independence in explaining variability among plasma cortisol, glucose and potassium concentrations in yellowfin bream (*Acanthopagrus australis*).

	Cortisol				Glucose				Potassium			
	All trawl	T_0_ trawl	T_120_ trawl	T_120_ all	All trawl	T_0_ trawl	T_120_ trawl	T_120_ all	All trawl	T_0_ trawl	T_120_ trawl	T_120_ all
**Treatment of fish**	-	-	-	**	-	-	-	○	-	-	-	○
**Sample time (T)**	**	-	-	-	**	-	-	-	**	-	-	-
**Sorting method (M)**	○	○	○	-	○	○	○	-	○	○	○	-
**Sorting delay (D)**	○	○	○	-	○	○	○	-	○	○	○	-
**M × D**	○	○	○	-	○	○	○	-	○	○	○	-
**T × M × D**	○	-	-	-	○	-	-	-	○	-	-	-
**Air exposure (AE)**	○	○	○	-	○	*	○	-	○	○	○	-
**AE × M**	○	○	○	-	○	○	○	-	○	○	○	-
**Air temperature (AT)**	○	○	○	-	○	*	**	-	○	○	○	-
**AT × M**	○	○	○	-	○	○	○	-	**	*	○	-
**Jellyfish weight**	○	○	○	-	○	○	○	-	*	*	○	-
**Cloud cover**	○	○	○	-	○	○	○	-	○	○	○	-
**Fish total length**	○	○	○	○	○	○	○	○	○	○	○	○
**River salinity**	*	○	*	-	○	○	○	-	○	○	○	-
**River temperature**	○	○	○	-	○	○	○	-	○	○	*	-

For each blood parameter, three models were applied to the trawled fish only (all individuals, and then just those sampled immediately after sorting—T_0_ and then after five days in cages—T_120_), and a fourth model applied to trawled (from conventional and water sorting) and control fish at T_120_ (defined as ‘treatment of fish’). -, term not appropriate in the model. The river salinity and temperature were bottom readings.

○ *p* > 0.05;

*p < 0.05;

**p < 0.01.

### Glucose

Like for cortisol, when considered across all trawled-and-discarded yellowfin bream, plasma glucose concentrations were much greater in T_0_ individuals (4.6 ± 0.2 mmol/L) than those at T_120_ (2.3 ± 0.1 mmol/L; LMM, *p* < 0.01). For just the trawled fish sampled at T_0_, glucose concentrations were positively and negatively associated with air exposure and temperature, respectively (LMM, *p* < 0.05; Table 3). But at T_120_, the reverse occurred for air temperature; manifesting as a positive association with glucose (LMM, *p* < 0.05; Table 3). There were no significant differences in plasma glucose among control (2.3 ± 0.1 mmol/L) and wet- (2.2 ± 0.1 mmol/L) or dry-sorted (2.4 ± 0.1 mmol/L) yellowfin bream at T_120_ (LMM, *p* > 0.05; Table 3).

### Potassium

Sample time had a significant effect on plasma potassium among all trawled-and-discarded yellowfin bream, with greater concentrations at T_0_ (7.1 ± 0.3 mmol/L) than at T_120_ (5.3 ± 0.1 mmol/L) (LMM, *p* < 0.01; Table 3). Further, among all trawled fish and those sampled only at T_0_, there were positive and negative associations between plasma potassium and the (i) weight of jellyfish in the trawl, and (ii) air temperature in dry-sorted fish only (i.e. air temperature × method interaction), respectively (LMM, *p* < 0.05; Table 3). For trawled fish at T_120_, potassium concentrations were negatively associated with river bottom temperature (LMM, *p* < 0.05; Table 2). Plasma potassium was not significantly different among control (4.9 ± 0.1 mmol/L) or wet- (5.2 ± 0.3 mmol/L) or dry-sorted (5.4 ± 0.2 mmol/L) yellowfin bream at T_120_ (LMM, *p* > 0.05; Table 3).

## Discussion

The results from this study indicate that being caught and discarded by penaeid trawls evokes a considerable acute stress response in yellowfin bream with plasma cortisol concentrations among T_0_ fish comparable to the mean range of peak post-stress levels observed for >10 species (average of 208 ng/mL) after similar capture-related stimuli (e.g. including approx. 30 min of angling, confinement, trawling, gillnetting or translocation) [33]. Further, while there are few other relevant data for yellowfin bream, the T_0_ cortisol concentrations were greater than those for their conspecifics post angling (3.6 ± 2.0 to 8.3 ± 2.0 ng/mL) [29] and other sparids such as pink snapper after 5-min trawls (reaching a peak of 42 ng/mL) [34] or red seabream (*Pagrus major*) after being hooked (12.2 ± 6.7 to 104.1 ±34.8 ng/mL) or trammel netted (12.2 ± 6.7 to 124.0 ± 47.3 ng/mL) [35].

While it is not possible to entirely partition causality among the observed physiological responses, the lack of any significant predictors of elevated cortisol, combined with few handling-related factors explaining the glucose and potassium response among T_0_ fish suggests that trawling, rather than sorting and discarding, evoked the most stress. The deleterious contribution of trawling is further supported by the significant effects of (i) river salinity and temperature on either cortisol or potassium among T_120_ fish, and (ii) jellyfish on potassium in T_0_ individuals. However, although neither the general sorting method nor the delay were significant main predictors of physiological responses, it is clear that on-board handling was still important, with air temperature and exposure affecting either immediate or delayed plasma glucose and potassium concentrations.

The physiological observations for T_0_ fish can be considered entirely representative of trawling and immediate discarding, since fish were bled within 2 min of being secured; effectively limiting any handling bias [18, 36]. But those fish that were caged might have been either less stressed (due to shelter, an absence of predators and more optimal salinity) or more stressed (due to enforced diurnal variability in river temperature and salinity; and from being caged) than normal. Controls were used, and subjected to the same transport system (on-board the transporting dory to the monitoring site) and handling, although it is impossible to completely remove all confounding effects when wild animals are held in captivity without being fed, but disturbed daily for welfare monitoring. The potential for such effects is evidenced by the elevated level of cortisol among controls, which probably reflected disturbances during sampling—given that glucose concentrations were close to expected baselines [29]. Cortisol levels do not always return to baseline levels within periods of confinement (e.g. in pink snapper) [4].

Notwithstanding the possibility of some experimentally induced stress, the response to trawling remained dominant. During initial capture as fish were herded into the trawl, they would have been severely exercised, and inevitably interacted with netting panels and other catches; all of which would have had considerable energetic costs [37]. For those fish caught at the start of each 40-min deployment, such exercise could have evoked an acute catecholamine response. Possibly, such impacts were exacerbated in the presence of even slightly less than physiologically optimal environmental conditions, such as salinity and temperature. For example, the osmoregulatory function of cortisol may have been triggered among individuals that were caught at the relatively low salinities [38]. These effects have been demonstrated for gilthead sea bream (*Sparus aurata*) in their adaptation to hypoosmotic shock [39]. As another example, the protracted increase in potassium at T_120_ associated with cooler water during trawling could simply reflect temperature stress [17, 27].

A novel result, and one that is poorly documented in the peer-reviewed literature describing fishing discards, was the increase in potassium concentrations in the presence of jellyfish; which probably occurred during trawling (because affected yellowfin bream were sorted on the dry tray). While the exact mechanisms of jellyfish impacts on potassium remain unclear, previous studies have shown that venom can inhibit ion pumps or cause cytolysis which could increase the concentration of potassium ions in plasma [31, 40, 41]. Additional research is required to explore the potential for such a relationship. It is also important to consider that we only assessed 30-min deployments. Conventional fishing can extend to 60 min, which conceivably would cumulatively increase the stress effects of the variables discussed above [42].

Once yellowfin bream were landed on board, it is apparent that general sorting method and delay had fewer primary or secondary physiological repercussions but, as might be expected, air exposure (and subsequent hypoxia) and temperature explained some variability [17, 43]. The changes in plasma glucose in response to air exposure were confounded by temperature [44]. Nevertheless, previous studies have shown that irrespective of temperature, prolonged exposure and the onset of hypoxia results in hyperglycaemia and elevated glucose [43]. Similar results have been demonstrated for several trout species [45], and Atlantic cod [46] among others [16, 25].

It is also well established that rapid changes in temperature cause metabolic disturbances (e.g. <1°C difference within minutes) [18]. In this study, yellowfin bream were removed from relatively warm water (25.2 ± 1.9°C) and exposed to mostly cooler air (23.0 ± 3.4°C at T_0_). The physiological consequences of this displacement were reflected in the negative relationships between both glucose and potassium and air temperature. Likewise, fish that were caught and exposed to air during relatively warm days and then returned to colder water at the monitoring site may have been additionally stressed; possibly explaining the positive association between air temperature and plasma glucose of trawled fish at T_120_.

Irrespective of the mechanisms contributing towards the observed variations in physiological responses, the potential for at least some short-term, sub-lethal impacts may be sufficient to warrant resolution attempts to maximise fish welfare [7, 47]. As a starting point, the negative impacts of jellyfish during trawling can be considerably reduced simply by using mechanical-type bycatch reduction devices, which would preclude interactions in the codend [26]. Such a direct approach might not be possible for mitigating other deleterious factors, but these could be indirectly addressed through operational strategies. For example, by recognizing extremes in key environmental factors (e.g. salinity and temperature), it might be conceivable to reduce cumulative impacts simply by shortening deployments when river temperatures or salinities are low. Reducing deployment duration might provide fish with more energetic resources to negate the effects of other factors [1, 30]. Ultimately, this approach could minimise short-term physiological consequences.

The results of this study, and especially the plasma cortisol and glucose dynamics, suggest an acute stress response of yellowfin bream to trawling and discarding. It remains unclear to what extent such physiological impacts might affect the ability of the species to avoid other sources of unaccounted fishing mortality (e.g. predation or infection), especially in areas of high trawling activity where individuals are repeatedly caught and stressed. For example, Atlantic cod (*Gadus morhua*) and rainbow trout (*Oncorhynchus mykiss*) that were exposed to repeated acute capture stress in simulated trials produced, among other effects, more frequently abnormal larvae and smaller egg sizes and less viable offspring, respectively than unstressed controls [48–49]. Such impacts warrant assessment as part of future research investigating the relationship between stress and injury on growth and reproduction [50].

In the interim, minimising deployment durations to the shortest practical times, and quickly sorting catches in water-filled trays (to reduce environmental extremes) would provide yellowfin bream not only with the best chances of surviving [7], but also fewer sub-lethal impacts, and presumably any subsequent cascading effects. On a broader scale, if several encounters with fishing gear elicit acute stress responses repeatedly, fish may become chronically stressed with potential implications for recruitment. Considering such unaccounted consequences, fisheries managers should strive for regulating biomass extraction holistically. In Europe, a newly implemented ban on discards considers such ancillary measures including catch quotas and survival provisions.

## Supporting Information

S1 Dataset Cortisol, glucose and potassium concentrations of each yellowfin bream sampled at T_0_ and T_120_ (including controls). This dataset includes blood plasma concentrations of each fish together with treatment categories and environmental, technical and biological co-variates.
(XLSX)
